# Structural characterization and hypolipidemic activities of purified stigma maydis polysaccharides

**DOI:** 10.1002/fsn3.1123

**Published:** 2019-07-03

**Authors:** Wenwen Deng, Xia Yang, Yuan Zhu, Jiangnan Yu, Ximing Xu

**Affiliations:** ^1^ Department of Pharmaceutics, School of Pharmacy, and Center for Drug/Gene Delivery and Tissue Engineering Jiangsu University Zhenjiang China

**Keywords:** bile acid‐binding, hyperlipidemia, poloxamer 407, stigma maydis polysaccharide

## Abstract

This study aimed to investigate structural features and antihyperlipidemic effects of the stigma maydis polysaccharide, termed SMP‐1. This polysaccharide was composed of D‐mannose, L‐rhamnose, D‐glucose, D‐galactose, L‐arabinose, D‐xylose, and D‐galacturonic acid, with a molar ratio of 1.00:0.21:1.41:1.44:0.70:0.44:0.56. The SMP‐1 was mainly bonded by (1 → 6) and (1 → 3) linkages, with various monosaccharides being evenly distributed in the main and side chains. Moreover, SMP‐1 had neither triple‐helical structure nor molecular aggregation. Importantly, the SMP‐1 could effectively bind the bile acids in vitro and significantly lower the total cholesterol, triglyceride, low‐density lipoprotein cholesterol levels, and moderately increase the high‐density lipoprotein cholesterol level in poloxamer 407‐induced hyperlipidemic mice. Moreover, pretreatment with SMP‐1 (≥300 mg/kg) could remarkably reduce fat accumulation and restore hepatocyte morphology in the liver of hyperlipidemic mice. Altogether, these findings indicated that SMP‐1 could be developed as a safe and effective food supplement for preventing and treating hyperlipidemic disorders.

## 1 INTRODUCTION

Hyperlipidemia is a systemic metabolic disorder that is usually caused by unhealthy diet intake. It is characterized by abnormally elevated levels of total cholesterol (TC), triglyceride (TG), low‐density lipoprotein cholesterol (LDL‐c), but decreased concentration of high‐density lipoprotein cholesterol (HDL‐c) in serum (Mao et al., 2017; Omari‐Siaw, Wang, et al., 2016). Hyperlipidemia is closely associated with many diseases, such as cardiovascular diseases (Omari‐Siaw, Zhu, et al., 2016), fatty liver (Leutner et al., 2017), and diabetes mellitus (Liu, Sun, Rao, Su, & Yang, 2013). Thus, this disorder has been considered as a severe threat to human health and life. Nowadays, statins and fibrates are the well‐known hypolipidemic drugs that are used to prevent cardiovascular diseases. However, these currently used hypolipidemic drugs often trigger several adverse effects such as hyperuricemia, peripheral neuropathy, memory loss, muscle damage, and skin rashes (Wu et al., 2016), especially in a long‐term treatment. Therefore, it is imperative to develop a safe and effective alternative for the treatment of hypolipidemic.

Recently, natural polysaccharides have attracted much attention in the field of biochemistry and pharmacology because of their health benefits such as immune regulation (Li et al., 2015; Surin, Surayot, Seesuriyachan, You, & Phimolsiripol, 2017), antitumor (Meng, Liang, & Luo, 2016; Zhao et al., 2013), antifatigue (Jia et al., 2014), antioxidation (Chaouch, Hafsa, Rihouey, Cerf, & Majdoub, 2016; Mao et al., 2014), antidiabetes (Liu, Sun, Rao, Su, Li, et al., 2013), antihyperglycemia, and antihyperlipidemia (Liu, Sun, Rao, Su, & Yang, 2013). Stigma maydis (corn silk), a by‐product from corn cultivation and processing (Žilić, Janković, Basić, Vančetović, & Maksimović, 2016), has been used worldwide as a traditional herbal medicine for the treatment of chronic diseases, such as diabetes (Pan et al., 2017), obesity (Lee et al., 2017), hypertension (George & Idu, 2015), and cystitis (Peng, Zhang, & Zhou, 2016), due to its multiple bioactive components including polysaccharides, flavonoids, steroids, alkaloids, saponins, proteins, volatile oils, tannins, and vitamins (Zhao et al., 2017). As one of the major ingredients in stigma maydis, stigma maydis polysaccharides account for many pharmacological activities with no toxicity (Zhao et al., 2017); however, its antihyperlipidemia effect is rarely reported.

In this study, a water‐soluble stigma maydis polysaccharide (SMP‐1) was isolated by the hot water extracting‐alcohol precipitating method, followed by purification with D315 anion exchange resin and Sephadex G‐100 resin successively. The purified SMP‐1 was then characterized by a series of analyses including high‐performance gel permeation chromatography (HPGPC), Fourier transform infrared (FT‐IR) spectrophotometry, precolumn derivatization high‐performance liquid chromatography, periodate oxidation analysis, partial acid hydrolysis, atomic force microscope (AFM), and Congo red staining. Afterward, the antihyperlipidemia activity of SMP‐1 was evaluated via testing bile acid‐binding capacity in vitro and serum lipid‐lowering effects in vivo. Collectively, this study would provide a solid evidence for the antihyperlipidemic effects of the stigma maydis polysaccharide, thus offering a safe and effective functional food supplement for the treatment of hyperlipidemia and other associated chronic diseases.

## 2 MATERIALS AND METHODS

### 2.1 Materials and chemicals

Stigma maydis was purchased from the local market (Zhenjiang, China). D315 macroporous resin and Sephadex G‐100 were purchased from Jiangsu Synthgene Biotechnology Co., Ltd. (Nanjing, China). Taurocholic acid sodium salt (purity >95%), glycodeoxycholic acid sodium salt (purity >95%), and other standard substances (purity >99%) were purchased from Aladdin Industrial Corporation. Test kits used for the determination of TC, HDL‐c, LDL‐c, and TG were purchased from Nanjing JianCheng Bioengineering Institute. All other chemicals and reagents were purchased from Sinopharm Chemical Reagent Co. Ltd. and they were of analytical grade.

### 2.2 Experimental animals

Male ICR mice (18–22 g) were obtained from the Laboratory Animal Center of Jiangsu University, with animal quality certificate number: SCXK (Su) 2013‐0011. The animals were housed in cages for one week to adapt to the environmental conditions with free access to laboratory food and water before experiment. The experimental protocol was approved by the Institution of Animal Ethical Committee, and the study was carried out in accordance with the National Institute of Health Guide for Care and Use of Laboratory Animals.

### 2.3 Extraction and purification of stigma maydis polysaccharide

The stigma maydis was extracted with hot water according to previous studies (Deng et al., 2012; Wang, Tian, et al., 2017). Then, the trichloroacetic acid solution (20%, 1:1 v/v) was added to the extracted solution to remove free proteins. The deproteinized solution was dialyzed in a dialysis bag (cut‐off molecular weight, 3,500 Da) against running water for 72 hr. After lyophilization, the crude stigma maydis polysaccharide was obtained.

The crude stigma maydis polysaccharide (5 g) was dissolved in distilled water and further purified using D315 macroporous resin (*D* 40 cm × 100 cm), with gradient eluting by 0, 0.1, 0.3, and 0.5 M of NaCl solution (500 ml each), respectively. The fraction eluted with distilled water was collected for another round of purification with the Sephadex G‐100 resin (*D* 24 cm × 80 cm). The distilled water was used as the eluent to produce a homogeneous fraction of the stigma maydis polysaccharide, SMP‐1. After that, the SMP‐1 fraction was collected, concentrated, dialyzed, and lyophilized. The resulting SMP‐1 was kept in a sealed container with a drying agent for the subsequent experiments.

### 2.4 Characterization of SMP‐1

#### 2.4.1 Chemical analysis

The carbohydrate content of SMP‐1 was determined with phenol‐sulfuric acid method using D‐glucose as the standard (Dubois, Gilles, Hamilton, Rebers, & Smith, 1951). The carbazole‐sulfuric acid spectrophotometric method was used to determine the uronic acid contents, with D‐Galacturonic acid as the standard. The Bradford method was also used to investigate the protein content of SMP‐1 using bovine serum albumin as the standard (Shi et al., 2015).

#### 2.4.2 Molecular weight determination

The molecular weight (Mw) of SMP‐1 was measured using the HPGPC method (Wang et al., 2011) equipped with a high‐performance liquid chromatography instrument (Agilent 1260), a TSK‐guard column PWH (7.5 × 75 mm), a TSK‐gel G4000PW (7.5 × 300 mm), and an evaporative light scattering detector (ELSD 1260). A 20 μl of the SMP‐1 solution (1 mg/ml) was eluted by the ammonium acetate solution (20 mM) at a flow rate of 0.6 ml/min. The calibration curve was obtained from the different standard dextrans with their respective Mw of 670, 270, 80, 25, and 5 kDa. The molecular weight of SMP‐1 was obtained through the regression equation of the molecular weights on a ln scale versus the retention time.

#### 2.4.3 UV and FT‐IR analyses

Powdered SMP‐1 (1 mg) was dissolved in distilled water. The UV spectrum was recorded within the wavelength ranging from 200 to 800 nm on a UV‐1700.

For FT‐IR analysis, completely dried SMP‐1 (1 mg) was mixed with suitable amount of KBr, ground and pressed into a transparent pellet. The FT‐IR spectrum of the sample was then recorded on a Bruker TENSOR 27 spectrometer (Bruker Optik).

#### 2.4.4 Analysis of monosaccharide composition

Monosaccharide composition was measured using 1‐phenyl‐3‐methyl‐5‐pyrazolone (PMP) precolumn derivatization HPLC method (Yang, Li, Xue, Wang, & Liu, 2014) with some modifications. Briefly, 5 mg of the powdered SMP‐1 was hydrolyzed with 1 ml of 2 M trifluoroacetic acid (TFA) at 110°C for 6 hr. NaOH (0.3 M) was then used to neutralize the excess acid. Afterward, PMP (0.5 M, as test reagent), lactose (10 mg/ml, as internal standard), and 250 μl of NaOH solution (0.3 M) were added to the hydrolysates and mixed using a vortex. The following reaction took place under the alkaline condition at 70°C for 30 min. After the reaction finished, the mixture was neutralized with HCl solution (0.3 M) and extracted thrice with trichloromethane. The water phase was collected for HPLC. Standard monosaccharides including D‐mannose (Man), L‐rhamnose (Rha), D‐(+)‐glucose (Glc), D‐galactose (Gal), L‐arabinose (Ara), and D‐(+)‐xylose (Xyl), as well as D‐galacturonic acid (GalA), were processed as the same procedure. The samples were analyzed using HPLC equipped with an Agilent 1260 HPLC system, an Agilent Eclipse XDB‐C18 column (150 mm × 4.6 mm, 5 μm), and a DAD detector (254 nm). The mobile phase consisted of A (0.1% formic acid with ammonium acetate aqueous solution), B (0.1% formic acid aqueous solution), and C (acetonitrile), with a gradient elution program: 0–6 min, A (80%) and C (20%); 6–6.5 min, B (80%) and C (20%); 6.5–20 min, B (80%) and C (20%).

#### 2.4.5 Periodate oxidation

The periodate oxidation that is usually performed to determine the glycosidic linkage position of polysaccharides was conducted according to a previous study (Ren et al., 2017). In brief, the SMP‐1 (10 mg) was dissolved in distilled water (<30 ml), to which the potassium periodate (KIO_4_) solution (15 mM) was added to a total volume of 30 ml. The mixture reacted at the room temperature (~26°C) under continuous stirring in a dark room for 8 days. An aliquot (0.1 ml) of the reaction solution was collected every 12 hr for the UV measurement at a wavelength of 223 nm, until the optical density value became stable. An equal volume (0.1 ml) of distilled water was added to the reaction system after each sample collection. Glycol (2 ml) was added to the mixture to terminate the reaction. The periodate consumption was determined using the calibration curve (*y* = 0.0404*x* + 0.0191, *R* ^2^ = 0.9981, where *x* represents the concentration of potassium periodate solution and *y* is the absorption value at 223 nm) of the different standard KIO_4_ solutions (0, 8, 16, 24, 32, 48, 64 mM). Then, 10 ml of the resultant solution was mixed with phenolphthalein indicator to titrate the amount of formic acid against NaOH solution (0.01 M).

#### 2.4.6 Partial acid hydrolysis

The partial acid hydrolysis was carried out using the method described in an earlier report (Song et al., 2017). The TFA has been previously reported to partially hydrolyze the polysaccharides and remove branch chains (Wang et al., 2015). Briefly, the SMP‐1 (25 mg) was hydrolyzed with 5 ml of TFA (0.05 M) at 100°C for 10 hr. Excess acid was removed using a vacuum evaporator. Then, the residues were dialyzed (cut‐off molecular weight, 3,500 Da) against distilled water for 72 hr. The nondialyzable fraction (inside of the bag, SMP‐1^a^) and dialysate (outside of the bag, SMP‐1^b^) were collected, concentrated, and freeze‐dried, respectively. The resulting fractions were then processed for monosaccharide composition analysis according to the procedures described in the Section 2.4.4.

#### 2.4.7 Congo red assay

Congo red method is usually performed to determine the triple‐helix structure of the polysaccharides (Chen, Zhang, Gao, Huang, & Wu, 2017). In brief, 2.0 ml of the SMP‐1 solution (1 mg/ml) was mixed thoroughly with 2.0 ml of Congo red reagent (100 μM). Then, the NaOH solution (2 M) was gradually added to the mixture to obtain a series of NaOH concentrations (0.05, 0.1, 0.2, 0.3, 0.4, and 0.5 M). At each NaOH concentration, the maximum absorption of the mixture was measured with ultraviolet‐visible scanning (300–700 nm), using the distilled water as control.

#### 2.4.8 Atomic force microscopy

Atomic force microscopy is employed to visualize the topographies of polysaccharides (Wang, Zhang, et al., 2017). Briefly, SMP‐1 was dissolved in ultrapure water and filtered through 0.45 μm membrane filter. The filtrate (5 μg/ml) was placed on a freshly cleaved mica disk and dried in ambient air for 4–8 hr at room temperature. The AFM images were obtained using a MFP‐3D (Asylum Research) microscope in tapping mode with commercial RTESP antimony doped Si tips (Bruker). The images were analyzed using NanoScope analysis software.

### 2.5 Evaluation of bile acid‐binding capacity in vitro

The potential bile acid‐binding capacity of SMP‐1 was measured according to previously described procedure with slight modification (Camire & Dougherty, 2003). Two main in vitro existing forms of bile acid (taurocholic acid sodium salt and glycodeoxycholic acid sodium salt) were used to detect the bile acid‐binding property of SMP‐1. Three groups were set: cholestyramine positive control (A), polysaccharide control group (B), and polysaccharide experimental group (C). The reaction time used for the quantity‐effect relation test with different concentrations of SMP‐1 (5, 10, 15, and 20 mg/ml) was 30 min. The concentration of SMP‐1 used for time‐response relation test with different time intervals (15, 30, 60, and 120 min) was 5 mg/ml. Afterward, the conjugation rate was determined as follows: Conjucationrate(%)=(C-B)/A×100%.

### 2.6 Treatment of poloxamer 407 induced hyperlipidemia

Poloxamer 407 (P407)‐induced acute hyperlipidemic mice have been proven to be an effective model for evaluating potential antihyperlipidemic compounds (Korolenko et al., 2016). A single intraperitoneal (i.p.) injection of P407 at a dose of 300 mg/kg can readily produce significant hyperlipidemia within approximately 4 days (Omari‐Siaw, Wang, et al., 2016).

The mice were randomly divided into seven groups (*n* = 10 in each group): normal control group (NC); model control group (MC); positive control group 1 (PC‐1, 200 mg/kg of Fenofibrate); positive control group 2 (PC‐2, 200 mg/kg of Zhibituo tablets); low concentration of SMP‐1 (L‐SMP‐1, 100 mg/kg); moderate concentration of SMP‐1 (M‐SMP‐1, 300 mg/kg); and high concentration of SMP‐1 (H‐SMP‐1, 500 mg/kg). The mice in the NC and MC groups were treated with normal saline by oral gavage, and the ones in the other five groups were treated with the drug or sample described in each group by oral gavage. One hour later, animals in all groups (except NC) received a single i.p. injection of P‐407 dissolved in normal saline at a dose of 300 mg/kg. Twenty‐four hours after the P‐407 treatment, the mice were fasted for 12 hr with free access to water. Afterward, the blood samples were collected from the orbital vein into heparin‐coated tubes and centrifuged at 2,600 *g* for 10 min at 4°C to obtain serum samples.

### 2.7 Determination of serum lipid profiles

The serum lipid profiles including TC, TG, LDL‐c, and HDL‐c in serum were determined with commercial test kits using an Epoch Microplate Spectrophotometer (BioTek Instruments).

### 2.8 Histopathological analysis

After the blood samples were collected, the mice were sacrificed via cervical dislocation. The liver was removed from each mouse, washed twice with phosphate‐buffered saline (PBS, pH = 7.4), and fixed in 5% formaldehyde overnight at 4°C. The liver samples were then washed thrice with double distilled water and embedded in paraffin wax. Afterward, the samples were sectioned into slices (5‐μm thickness) using a rotary microtome (leica‐RM2235). The transverse sections were collected on glass slides, de‐paraffinized, and stained with hematoxylin and eosin (H&E; Beyotime Institute of Biology) and observed with an electron microscope (Nikon Eclipse 90i microscope) at 200× magnification.

### 2.9 Statistical analysis

All values were expressed as means ± standard deviation (*SD*) of three replicates. Statistical analyses were performed using SPSS 19.0 software (SPSS Inc.). The differences between the various groups were analyzed by one‐way analysis of variance (ANOVA) followed by Tukey's post hoc test. A *p*‐value of less than 0.05 or 0.01 was, respectively, considered as either statistically significant or very significant.

## 3 RESULTS AND DISCUSSION

### 3.1 Chemical components and molecular weight analysis

The carbohydrate content of SMP‐1 was 79.71 ± 2.07% according to the results of phenol‐sulfuric acid method. The contents of protein and uronic acid in SMP‐1 were 0.92 ± 0.09% and 14.56 ± 0.67%, respectively.

**Figure 1:**
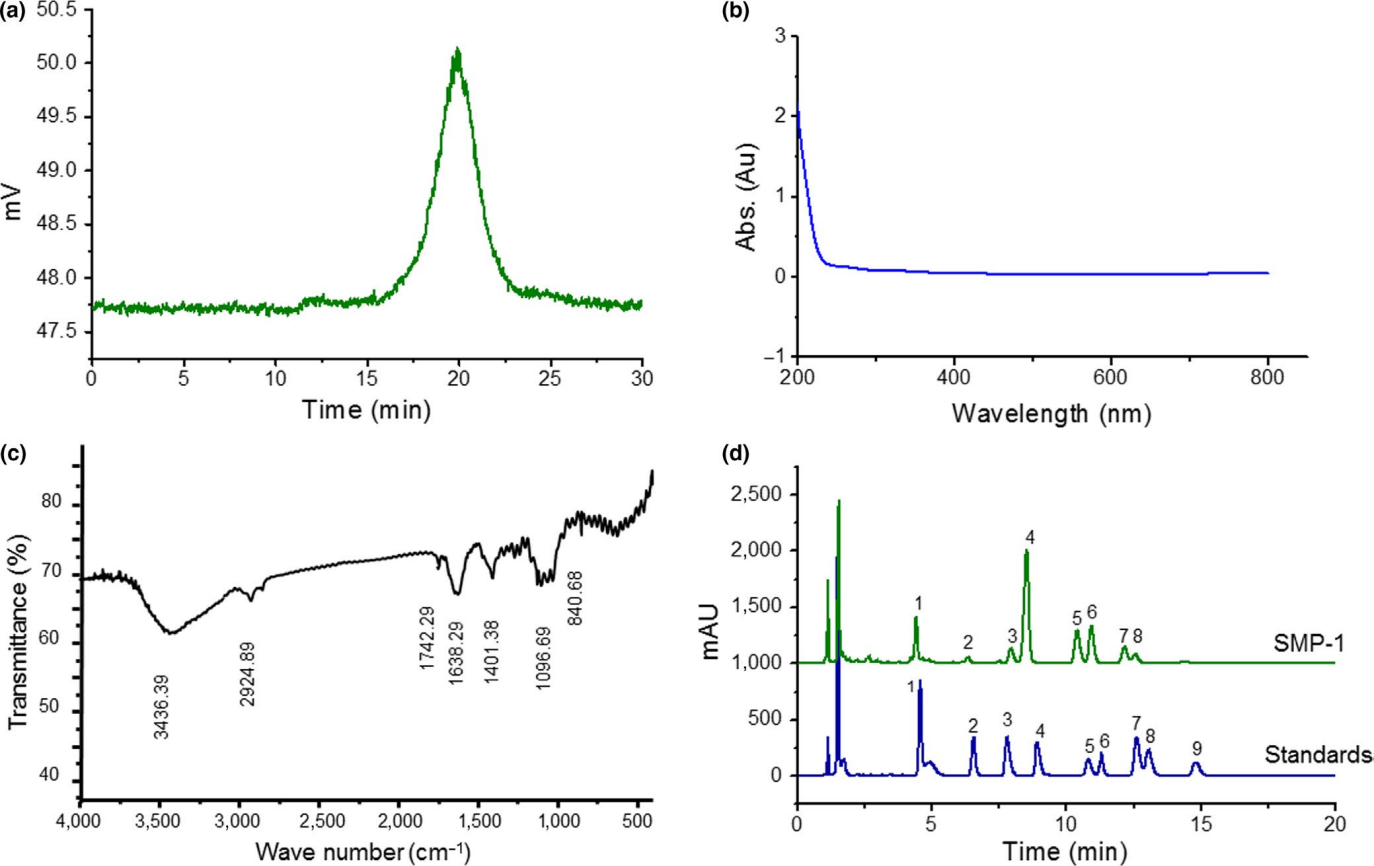
Characterization of SMP‐1. (a) The HPGPC chromatogram of SMP‐1, with the retention time of 19.88 min; (b) The UV spectrum of SMP‐1 within the wavelength from 200 to 800 nm; (c) The IR spectrum of SMP‐1 in the wave number region between 4,000 and 400 cm^−1^; (d) HPLC chromatograms of the monosaccharides (1. PMP; 2. D‐mannose; 3. L‐rhamnose; 4. D‐galacturonic acid; 5. lactose; 6. D‐glucose; 7. D‐galactose; 8. L‐arabinose; 9. D‐xylose; 10. L‐fucose) in the mixture of standard monosaccharides and the SMP‐1

The chromatogram recorded by HPGPC showed that SMP‐1 gave rise to a single symmetrical peak (Figure 1a), indicating that SMP‐1 was homogeneous. According to the calibration curve of ln*Mw* = −0.8035*t* + 25.441 (R^2^ = 0.995, where *Mw* was the molecular weight, and *t* was the retention time), the average Mw of SMP‐1 was about 12.93 kDa.

### 3.2 UV and FT‐IR analysis

The UV analysis demonstrated that there was almost no absorption at 260 or 280 nm (Figure 1b), suggesting the very low levels of nucleic acids and proteins in SMP‐1. This finding is consistent with the results of chemical component analysis.

The IR spectroscopy indicated that SMP‐1 possessed typical absorption peaks of polysaccharides in the range of 4000–400 cm^−1^ (Figure 1c). It showed broad and intense absorption bands at 3,436.39 and 2,924.89 cm^−1^, which were generated by the stretching vibration of O–H and C–H, respectively (Jin et al., 2018). The strong absorptions at 1,750–1,600 cm^−1^ could be attributed to the C=O stretching vibration of the carboxyl group (Ayyappan, Sundaraganesan, Aroulmoji, Murano, & Sebastian, 2010). The absorption peaks within the range of 1,100–1,010 cm^−1^ were attributed to the C–O stretching vibration of pyranose ring (Daemi & Barikani, 2012). Moreover, the characteristic absorption band at 840.68 cm^−1^ confirmed the existence of α‐configurations of sugar units (Chylinska, Szymanska‐Chargot, & Zdunek, 2016). Altogether, the IR spectrum indicates that SMP‐1 is likely to possess sugar units with pyranose rings and α‐configurations.

### 3.3 Monosaccharide composition analysis

The results of precolumn derivatization HPLC showed that the monosaccharide composition of SMP‐1 hydrolysate contained Man, Rha, Glc, Gal, Ara, and Xyl, as well as GalA, with a relative molar ratio of 1.00:0.21:1.41:1.44:0.70:0.44:0.56 (Figure 1d). The monosaccharide composition of CPS was also consistent with the previous study (Zou et al., 2017) but with different molar ratios, which could be due to the differences during extraction and analysis.

### 3.4 Periodate oxidation

The position of glycosidic linkage of SMP‐1 was evaluated using the consumption of KIO_4_ and NaOH. The generated formic acid was titrated with NaOH (0.01 M). As a result, the sugar residue (1 M) of SMP‐1 consumed 0.81 M of KIO_4_, and 0.44 M of formic acid was produced per molar sugar residue. According to the principle of periodate oxidation (Q. Peng, Li, Xue, & Liu, 2014), these results indicate that the SMP‐1 residues are mainly bonded by (1 → 6) and (1 → 3) linkages with molar ratio of 1:1.47, and no (1 → 2) or (1 → 4) linkages are found in SMP‐1.

### 3.5 Partial acid hydrolysis

The monosaccharide compositions of the nondialyzable (SMP‐1^a^) and the dialyzable (SMP‐1^b^) fractions of SMP‐1 were separately analyzed. The results showed that both fractions of SMP‐1 were of the same set of monosaccharides (Man, Rha, Glc, Gal, Ara, Xyl, and GalA) with similar molar ratios (Table 1), suggesting that seven different types of monosaccharides are evenly distributed in the main and side chains of the polysaccharide (Song et al., 2017).

**Table 1:** The molar ratios in partial acid hydrolysis analysis of SMP‐1

Fractions	Molar ratios						
	Man	Rha	Glc	Gal	Ara	Xyl	GlcA
SMP‐1^a^	1.00	0.24 ± 0.02	0.81 ± 0.01	1.53 ± 0.06	0.31 ± 0.02	0.45 ± 0.01	0.17 ± 0.00
SMP‐1^b^	1.00	0.15 ± 0.01	1.20 ± 0.04	1.90 ± 0.02	0.79 ± 0.01	0.87 ± 0.03	0.12 ± 0.02

a Dialyzable fraction.

b Nondialyzable fraction.

### 3.6 Congo red test

**Figure 2:**
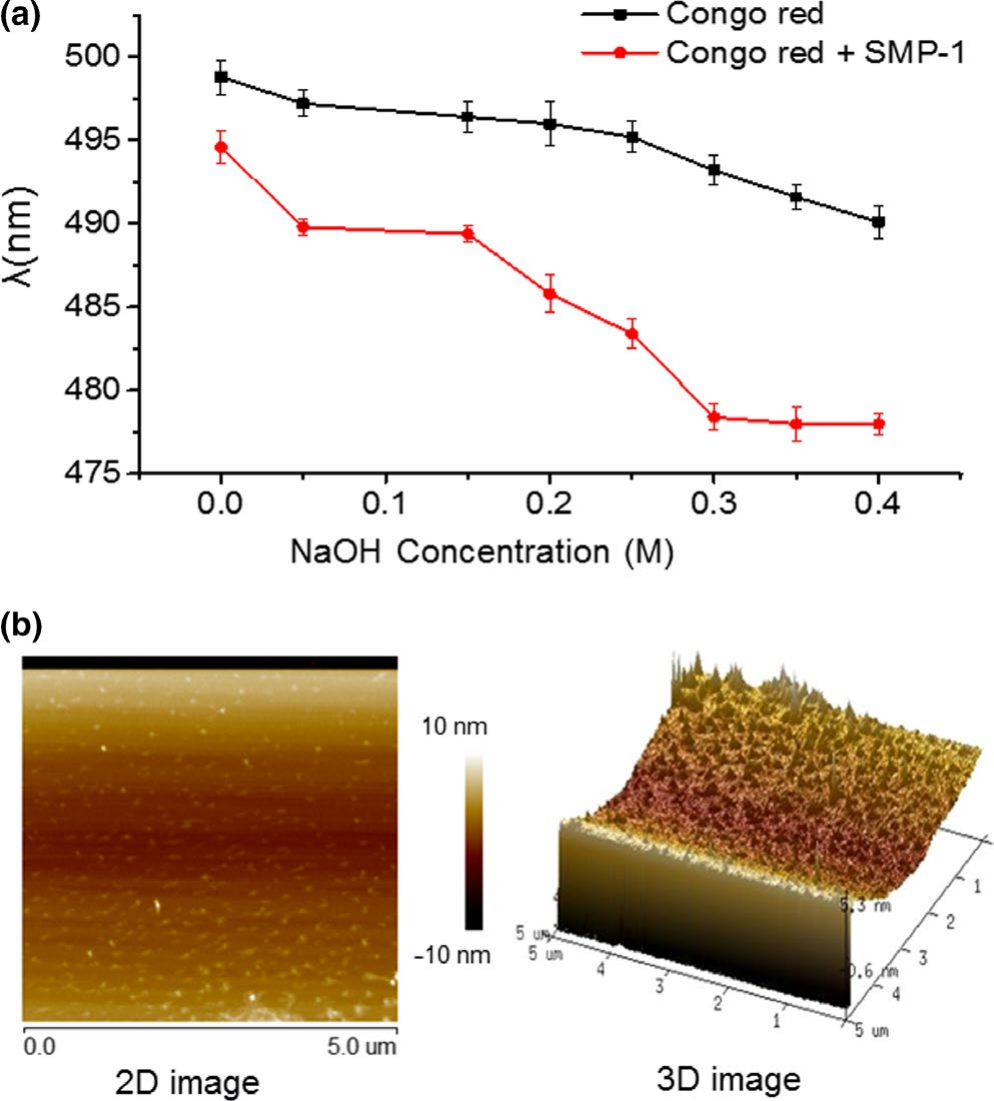
Chain conformation determination. (a) Changes in maximum absorption wavelength of Conge red and Congo red‐SMP‐1 complex; (b) AFM images of SMP‐1 at the concentration of 5 μg/ml: two‐dimensional (2D) image of SMP‐1 (left) and two‐dimensional (3D) image of SMP‐1 (right)

As previously reported, the polysaccharides with a helical conformation could give rise to a bathochromic shift of the maximum absorption λ of the Congo red–polysaccharide complex, in comparison with pure Congo red (Xu et al., 2018). The absorptions of Congo red‐SMP‐1 complex and pure Congo red were determined at various NaOH concentrations. As shown in Figure 2a, the maximum absorption λ of the Congo red‐SMP‐1 complex was similar to that of the pure Congo red, which decreased in tandem with the increase of NaOH concentrations. This finding suggests that the conformation of SMP‐1 in solution is not triple‐helical.

### 3.7 Atomic force microscopy

Atomic force microscopy is a powerful tool used to detect the surface topology and local mechanical properties of biological molecules (Li et al., 1999). Figure 2b shows the 2D and 3D images of SMP‐1. The images revealed evenly distributed sharp peak‐shaped structures with the height ranging from 0.6–5.3 nm. This result indicates that SMP‐1 has almost no molecular aggregation.

### 3.8 In vitro bile acid binding

Some components of food and herbs conjugate with bile acid and prevent its absorption, thereby producing certain hypolipidemic effects (Kahlon, Chapman, & Smith, 2007a, 2007b). Bile acid exists in the form of cholates such as taurocholate and glycocholate, and therefore, the capacity to bind cholates can be used to evaluate the hypolipidemic activities of active components in vitro.

**Figure 3:**
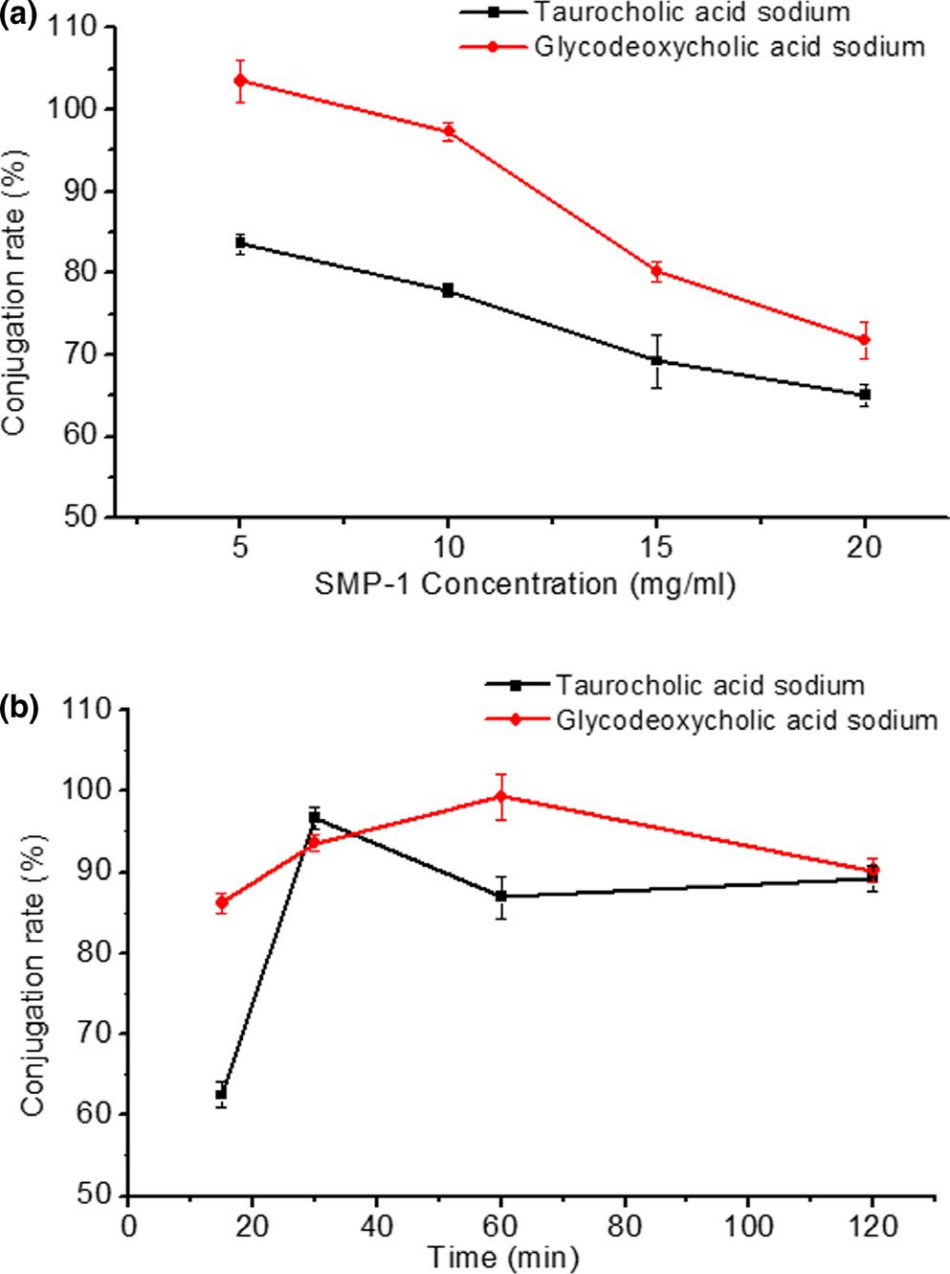
In vitro bile acid‐binding assay. (a) The conjugation rates with cholates (taurocholic acid sodium and glycodeoxycholic acid sodium) varied at different SMP‐1 concentrations (5, 10, 15, and 20 mg/ml); (b) The conjugation rates with cholates (taurocholic acid sodium and glycodeoxycholic acid sodium) changed with the extension of reaction time (15, 30, 60, and 120 min)

The bile acid conjugation rate of SMP‐1 was evaluated using the SMP‐1 concentration and the reaction time as the variable, respectively. As shown in Figure 3a, when different concentrations of SMP‐1 (5, 10, 15, 20 mg/ml) reacted with the cholates for 30 min, the SMP‐1 showed a strong binding capacity to both taurocholic acid sodium (65.96%–83.64%) and glycodeoxycholic acid sodium (71.76%–103.50%). Interestingly, as the SMP‐1 concentration increased, the cholate‐binding capacity decreased in general. This observation was consistent with previous reports (Gong et al., 2015; Kim & White, 2010) which have shown that polysaccharides with smaller Mw and lower viscosity of solution can have stronger bile acid‐binding in vitro. In this study, SMP‐1 with lower concentration probably had lower viscosity, thus exhibiting greater bile acid‐binding rates. Figure 3b displays the time–response relation when the SMP‐1 concentration was 5 mg/ml. Although irregular cholates‐conjugation profiles were observed with the extension of the reaction time from 15 to 120 min, the highest conjugation rate (~100%) of both cholates (taurocholic acid sodium and glycodeoxycholic acid sodium) was attained within an hour. Altogether, these results demonstrate that SMP‐1 possesses an excellent performance on binding bile acid, suggesting its hypolipidemic potential.

### 3.9 In vivo hypolipidemic activities

The serum lipid profiles (TC, TG, LDL‐c, and HDL‐c in serum) are shown in Table 2. There was a remarkable (*p* < 0.01 or *p* < 0.05) elevation in serum TC, TG, and LDL‐c, but a significant (*p* < 0.05) decrease in serum HDL‐c in the MC group when compared to the NC group, indicating the successful establishment of the hyperlipidemic mice model. Mice that were pretreated with positive control drugs (Fenofibrate and Zhibituo tablets) and SMP‐1 with moderate and high concentrations (M‐SMP‐1 and H‐SMP‐1) showed significantly (*p* < 0.01 or *p* < 0.05) decreased serum levels of TC, TG, and LDL‐c and moderately increased HDL‐c level in comparison with the animals in the MC group. Notably, pretreatment with H‐SMP‐1 could lower the levels of TC and TG in the hyperlipidemic mice to a level similar to that of the positive controls. These findings collectively confirm the therapeutic efficacy of SMP‐1 in the decreasing serum TC, TG, and LDL‐c levels of the hyperlipidemic mice.

**Table 2:** Effect of SMP‐1 on serum lipid profile levels in P407‐induced hyperlipidemic mice

Groups	Serum lipids			
	TC (mmol/L)	TG (mmol/L)	LDL‐C (mmol/L)	HDL‐C (mmol/L)
NC	3.55 ± 0.42	0.77 ± 0.05	0.66 ± 0.13	4.05 ± 0.72
MC	4.40 ± 0.51^††^	0.89 ± 0.05^††^	0.92 ± 0.23^†^	3.26 ± 0.42^†^
L‐SMP‐1	3.87 ± 0.32*	0.82 ± 0.09	0.75 ± 0.21	3.69 ± 0.84
M‐SMP‐1	3.62 ± 0.24*	0.61 ± 0.22**	0.52 ± 0.09**	3.71 ± 0.35
H‐SMP‐1	3.43 ± 0.80**	0.45 ± 0.12**	0.58 ± 0.18**	3.52 ± 0.31
PC‐1	3.36 ± 0.26**	0.73 ± 0.12*	0.53 ± 0.20**	3.36 ± 0.31
PC‐2	3.40 ± 0.78**	0.52 ± 0.20**	0.54 ± 0.23*	3.55 ± 0.41

Note

PC‐1 and PC‐2 represent Fenofibrate and Zhibituo tablets, respectively.

Values are mean ± *SD*; *n* = 7 serum samples from seven mice.

* *p* < 0.05 versus Model control (MC) group;

** *p* < 0.01 versus Model control (MC) group;

^†^ *p* < 0.05 versus Normal control (NC) group;

^††^ *p* < 0.01 versus Normal control (NC) group.

Based on the results of the acid‐binding assay, one possible antihyperlipidemic mechanisms of SMP‐1 is the bile acid sequestration (Liu et al., 2012). The SMP‐1 can act as a sequestrant of the bile acids in the small intestine to reduce the reabsorption and increase the fecal excretion of bile acids, which further stimulate the bile acid synthesis to keep a constant level of bile acids in liver. Bile acid synthesis requires consumption of a large quantity of cholesterol, thus lowering the LDL‐c in the blood.

### 3.10 Histopathological analysis

**Figure 4:**
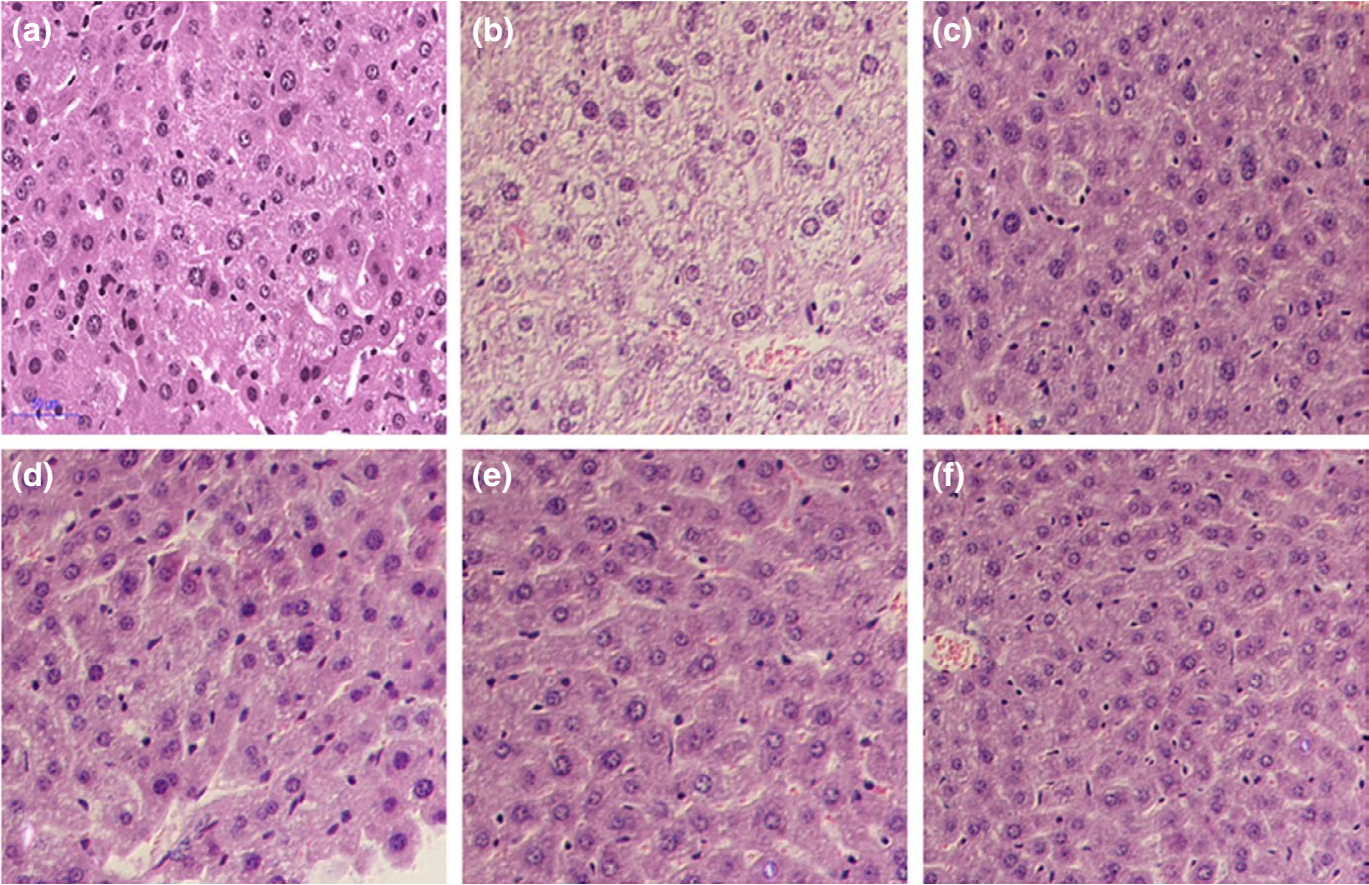
Histopathological photomicrographs of mice liver tissues. (a) Normal control group (NC); (b) Model control group (MC); (c) Positive control group 1 (PC‐1, 200 mg/kg of Fenofibrate); (d) Low concentration of SMP‐1 (L‐SMP‐1, 100 mg/kg); (e) Moderate concentration of SMP‐1 (M‐SMP‐1, 300 mg/kg); (f) High concentration of SMP‐1 (H‐SMP‐1, 500 mg/kg)

To investigate the beneficial effects of SMP‐1 on liver, the main organ involved in lipid metabolism, the histopathological analysis was performed. As shown in Figure 4, there was a large amount of fat droplets accumulated in the liver of the mice in MC group (Figure 4b) compared with the NC group (Figure 4a). The livers of mice pretreated with SMP‐1 at low concentration (L‐SMP‐1) showed an obvious improvement (Figure 4d) of hepatocyte morphology compared to the MC group. Moreover, the livers of mice pretreated with M‐SMP‐1 (Figure 4e) and H‐SMP‐1 (Figure 4f) displayed significantly reduced accumulation of fat droplets compared to the MC group, with the hepatocyte morphology close to that of the PC‐1 (Figure 4c) and NC group (Figure 4a). This result further supports the effective hypolipidemic activity of SMP‐1.

### 3.11 Structure‐function relationship

As aforementioned, SMP‐1 was mainly composed of Gal (25%), Glc (24.5%), and Man (17.4%), followed by Ara (12.2%), GalA (9.7%), Xyl (7.6%), and Rha (3.6%). The monosaccharide composition of SMP‐1 was similar to that of the previously reported Ginkgo biloba leaf polysaccharide which ameliorated changes in the lipid profile in serum and liver tissue of rats with nonalcoholic fatty liver disease (Yan et al., 2015). In addition, SMP‐1 contained a relatively high content of GalA, a major component of pectin that is well known for its hypolipidemic function (Ridley, O'Neill, & Mohnen, 2001; Vergarajimenez, Conde, Erickson, & Fernandez, 1998). In a word, the hypolipidemic activity of SMP‐1 is probably due to its monosaccharide components that are known for blood lipid regulation. Further studies will be carried out to clarify other possible relationships between the structure of SMP‐1 and its hypolipidemic activity.

## 4 CONCLUSION

In this study, a water‐soluble polysaccharide from the stigma maydis (SMP‐1) was isolated, characterized, and investigated on its hypolipidemic activity in P407‐induced hyperlipidemic mice model. The SMP‐1 (average Mw = 12.93 kDa) was a homogeneous polysaccharide with no triple‐helical structure and no molecular aggregations in solution. Importantly, the SMP‐1 could effectively bind the bile acids in vitro and significantly lower the TC, TG, and LDL‐c levels and moderately increase the LDL‐c level in serum of the P407‐induced hyperlipidemic mice. Moreover, pretreatment with SMP‐1 (≥300 mg/kg) could remarkably reduce the fat accumulation in the liver of the hyperlipidemic mice. Collectively, these findings demonstrate that the stigma maydis polysaccharides can be developed as a safe and effective food supplement for preventing and treating the hyperlipidemia disorders.

## CONFLICT OF INTEREST

The authors declare that they do not have any conflict of interest.

## ETHICAL REVIEW

The experimental protocol was approved by the Institution of Animal Ethical Committee, and the study was carried out in accordance with the National Institute of Health Guide for Care and Use of Laboratory Animals.
